# Determinants of survival of people living with HIV/AIDS on antiretroviral therapy in Brazil 2006–2015

**DOI:** 10.1186/s12879-019-3844-3

**Published:** 2019-02-28

**Authors:** Tara D. Mangal, Mariana Veloso Meireles, Ana Roberta Pati Pascom, Ronaldo de Almeida Coelho, Adele Schwartz Benzaken, Timothy B. Hallett

**Affiliations:** 1 Medical Research Council Centre for Outbreak Analysis and Modelling, Department of Infectious Disease Epidemiology, School of Public Health, Imperial College London, Norfolk Place, London, W2 1PG UK; 2 Ministry of Health, Department of STI, HIV/AIDS and Viral Hepatitis, SRTVN Quadra 701, Lote D, Edifício PO700 CEP, Brasília, Distrito Federal 70719-040 Brazil

**Keywords:** HIV/AIDS, Mortality, Brazil, Antiretroviral treatment

## Abstract

**Background:**

We compared AIDS-related mortality rates in people living with HIV (PLHIV) starting antiretroviral therapy (ART) in Brazil during 2006–2015 and examined associated risk factors .

**Methods:**

Data on ART use in PLHIV and AIDS mortality in Brazil was analysed with piecewise constant exponential models. Mortality rates and hazard ratios were estimated for 0–6, 6–12, 13–24, 25–36 and > 36 months of ART use and adjusted for region, age, sex, baseline CD4 cell count and calendar year of ART initiation. An additional analysis restricted to those with data on risk group was also performed.

**Results:**

269,076 individuals were included in the analysis, 165,643 (62%) males and 103,433 (38%) females, with 1,783,305 person-years of follow-up time. 21,749 AIDS deaths were reported and 8898 deaths occurred in the first year of ART. The risk of death in the first six months decreased with early ART initiation; those starting treatment early with CD4 > 500 cells per μL had a hazard ratio of 0.06 (95% CI 0.05–0.07) compared with CD4 < 200 cells per μL. Older age, male sex, intravenous drug use and starting treatment in earlier calendar years were associated with higher mortality rates. People living in the North, Northeast and South of Brazil experienced significantly higher AIDS mortality rates than those in the Southeast (HR 1.44, [95% CI 1.35–1.54], 1.10 [1.05–1.16] and 1.22 [1.17–1.28] respectively).

**Conclusions:**

Early treatment is likely to have contributed to the improved survival in PLHIV on ART, with the greatest benefits observed in women, younger age-groups and those living in the North.

**Electronic supplementary material:**

The online version of this article (10.1186/s12879-019-3844-3) contains supplementary material, which is available to authorized users.

## Background

Estimation of mortality is a key indicator of the performance of an HIV programme, however few large studies estimate survival on ART in Latin American countries and those that include Brazil, tend to focus on cohorts often based in urban areas such as Rio de Janeiro. [1, 2] Extrapolating findings from smaller cohort studies in Brazil to a national level is complex and requires consideration of the wider social and economic contexts. [3–7] Similarly, aggregating national data can distort our understanding of the mortality profile of people living with HIV (PLHIV), since the majority of HIV/AIDS cases come from the Southeast, a densely populated and affluent region of the country. [8]

In 2017, 48,000 new HIV infections and 14,000 AIDS-related deaths were estimated in Brazil, corresponding, respectively, to 48 and 37% of the total incidence and AIDS-mortality reported in Latin America. [9] The country’s AIDS Programme has adopted a policy of universal access to ART, becoming the first middle-income country to provide free health care and treatment. Over the past decade, the HIV/AIDS programme has undergone rapid expansion with 585,000 PLHIV on ART by September 2018, [10] improving the quality of life and life expectancy of PLHIV and reducing the incidence of opportunistic infections. [11, 12] However, the modest reductions in reported mortality rates disguise significant regional heterogeneities. AIDS mortality rates in North, North-East and Central regions have been increasing over the past ten years (2006-2016) whilst two north-eastern states (Amapá and Pará) experienced increases of 105.7 and 103% respectively. [13, 14]

Evidence of disparities in health outcomes by sex, risk group and region in PLHIV have been documented in Brazil. [5, 6, 12, 13, 15, 16] Intravenous drug users (IDUs) may be less likely to access ART and tend to enter into care late, consequently experiencing higher mortality rates [15], although some studies have found no significant difference between mortality rates in IDUs compared with non-IDUs. [3, 4] Additionally, the hazard of an AIDS death is higher in men compared with women and in heterosexual men compared with men who have sex with men (MSM). [7, 12]

The 2016 World Health Organization guidelines recommending early ART initiation to all PLHIV irrespective of CD4 cell count were based on a growing body of evidence showing the increased risk of AIDS or death associated with delaying treatment. [17] In fact Brazil introduced treatment for all in 2014, following rising CD4 eligibility thresholds from 200 cells per μL until 2009, 350 cells per μL until 2012 and 500 cells per μL in 2013. [18] In addition to the individual benefits, early treatment can also reduce the risk of HIV transmission to partners [19–22], impacting incidence at the population level. [23, 24] The current evidence consists mainly of cohort studies or randomised control trials conducted under experimental conditions. In many settings, only those with severe illness began ART with high CD4 cell counts, therefore we cannot expect the mortality profile of these individuals to appropriately reflect that of otherwise healthy PLHIV starting ART early. These estimates may therefore not be generalizable to the overall population of PLHIV using ART.

Three surveillance databases in Brazil (notification of HIV/AIDS cases, CD4 cell counts, ART dispensations) have been linked with vital registration data to provide information on over one million PLHIV, tracking individuals from diagnosis, through laboratory tests, treatment programmes and death. Here we present an analysis of survival in PLHIV on ART in Brazil over the past ten years. We examine mortality rates at six to twelve-month intervals adjusting for potential risk factors using individual-level data acquired from routine case-reporting.

## Methods

### Study population

The data were collated by the Department of STI, HIV/AIDS and Viral Hepatitis (DIAHV) in the Ministry of Health, Brazil and individually linked via a probabilistic algorithm. [25] ART dispensations, CD4 cell counts and HIV/AIDS case-reports between January 2006 and September 2016 were collected by each administrative unit and sent to the DIAHV for cleaning, processing and linkage. AIDS mortality data were collected using the Mortality Information System database which uses the International Classification of Diseases, Tenth Revision (ICD-10) to classify causes of death. Data on deaths due to causes unrelated to AIDS were not available. Internal validation was performed, including additional follow-up when required. The DIAHV performed checks on the data, removed duplicate entries and then anonymised the data, assigning each entry a unique reference number. This procedure is performed annually by DIAHV and is described in detail elsewhere [15].

Individuals were included if they were aged ≥15 years at ART initiation with a CD4 cell count within three months prior to starting ART. We excluded those missing age and sex data and individuals starting ART in 2016 due to insufficient follow-up time. The primary endpoint was reported AIDS death (ICD-10 codes B20-B24). Individuals without recorded deaths were censored six months after the last CD4 cell count measurement or ART dispensation, or September 2016, whichever date occurred first. We assumed an intention-to-treat approach, ignoring changes in therapy or breaks in treatment programmes. Patients were lost to follow-up (LTFU) if they had no recorded clinical data (CD4 cell count measurements or ART dispensations) within one year of the database end date with no reported date of AIDS death and censored them six months after their last clinic visit. [26] Individuals who died of causes unrelated to AIDS would also be included in the LTFU estimates.

### Statistical analysis

A piecewise constant exponential regression model was used to estimate AIDS-mortality rates and hazard ratios (HR) for individuals on ART in Brazil. The baseline hazard varied allowing estimation of changing mortality rates by length of time on treatment (0–6, 7–12, 13–24, 25–36 and ≥ 36 months). Individual-level covariates at baseline (age, CD4 cell count and sex and region of residence [North, Northeast, Central, South and Southeast]) were included along with calendar year as fixed predictors. Individuals were classified into four states characterised by their CD4 cell count states at ART initiation (≥500, 350–499, 200–349 and < 200 cells per μL) using the last CD4 count reported before starting ART. All baseline covariates were tested using univariate analysis and retained if they showed both a significant association with mortality and subsequently improved the fit of the multivariate model. Mortality rates were assumed to be constant within each region and CD4 state. Confidence intervals around the estimates were derived from the model assuming normality.

Differences in the effects of early ART (starting treatment with CD4 cell counts ≥500 cells per μL) by region, age and sex were assessed by including interactions and the most parsimonious model was selected using Aikake’s Information Criterion with a stepwise deletion process.

A second survival analysis was separately conducted in which additional data regarding self-reported probable mode of transmission (MSM, bisexual, heterosexual, IDU) was included. Those with other modes of transmission (haemophiliac, blood transfusion, biological accident) or missing data were included as separate covariate groups and baseline characteristics were compared using Wilcoxon signed-rank tests (for age) and chi-square tests (sex and region). Model selection was performed as described above.

### Model validation

The model’s predictive ability was evaluated using repeated 10-fold cross-validation. Data were randomly split into training and testing sets containing 90 and 10% of observations respectively. The analysis was performed on the training set, validated on the testing set and accuracy (calculated as 1 – misclassification error) using the validation results was measured. This was repeated ten times and the average of the accuracy values was used as the estimate of the model’s performance.

A sensitivity analysis explored the impact of our assumptions on censoring, modifying the censoring date to one year after the last observation. Additionally, we expanded the analysis to include those with CD4 cell counts within six months prior to ART initiation. We also excluded those contributing less than one month of person-time on ART to check for systematic bias. Deaths in this group are more likely due to a late diagnosis and insufficient time on treatment. All analyses were performed using R version 3.1.1 (http://www.r-project.org).

## Results

### Summary of population

Of 473,628 records of persons initiating ART in Brazil during 2006–2015, 269,076 were eligible for inclusion in this analysis, 165,643 (62%) males and 103,433 (38%) females (Table 1). The majority were aged between 25 and 44 years (167,911 people, 62%) and 39% started ART with CD4 cell counts > 350 cells per μL. The median age at ART initiation decreased over time from 39 years (inter-quartile range [IQR] 33–46 years) in 2006 to 34 years (IQR 27–43 years) in 2015. Data on the region of residence were missing for 32 individuals (0.01%) and the majority were resident in the Southeast (129,531 people, 48%) and South regions (62,400 people, 23%). The median CD4 cell count at treatment initiation increased over time from 308 cells per μL in 2006 to 393 cells per μL in 2015.

**Table 1:** Baseline characteristics of people living with HIV at the time of starting ART. Data on risk group are available for 170,930 (63.5%) individuals. IDU – intravenous drug user; MSM – men who have sex with men

	Number (%)	Deaths (%)
Time on ART		
0–6 months	269,076 (100)	6836 (31)
7–12 months	259,734 (97)	2062 (9)
13–24 months	252,929 (94)	3116 (14)
25–36 months	223,556 (83)	2558 (12)
> 36 months	185,632 (69)	7177 (33)
Age (years)		
15–24	27,136 (10)	1238 (6)
25–34	84,444 (31)	5900 (27)
35–44	83,467 (31)	7285 (33)
> 45	74,029 (28)	7326 (34)
CD4 cell count (cells per μL)		
< 200	87,765 (33)	13,946 (66)
200–349	77,229 (29)	4685 (22)
350–499	49,911 (19)	1750 (8)
≥ 500	54,171 (20)	1368 (6)
Sex		
Male	165,643 (62)	14,536 (67)
Female	103,433 (38)	7213 (33)
Year of ART initiation		
2006	18,056 (7)	2530 (12)
2007	21,699 (8)	3015 (14)
2008	33,666 (13)	4068 (19)
2009	22,111 (8)	2710 (12)
2010	22,387 (8)	2342 (11)
2011	22,621 (8)	1967 (9)
2012	26,968 (10)	1911 (8)
2013	29,668 (11)	1466 (7)
2014	35,818 (13)	1092 (5)
2015	36,082 (13)	648 (3)
Region		
Southeast	129,531 (48)	9868 (45)
Central-West	17,261 (6)	1450 (7)
Northeast	41,684 (15)	3327 (15)
South	62,400 (23)	5633 (26)
North	18,168 (7)	1469 (7)
missing	32 (0.01)	2 (0.009)
Risk group		
Heterosexual male	51,069 (19)	5552 (26)
Bisexual male	11,244 (4)	880 (4)
MSM	36,198 (13)	1857 (9)
Male IDU	7393 (3)	1401 (6)
Female non-IDU	63,143 (23)	4910 (23)
Female IDU	1665 (0.6)	294 (1)
other	218 (0.08)	28 (0.1)
missing	98,146 (36)	6827 (31)
Total	269,076	21,749

The percentages in each subgroup refer to the proportion of individuals in each category of the subgroup. The numbers of individuals by time on treatment however, where individuals contribute to multiple periods of treatment time, begin at the total number at baseline (i.e. 100%) and decrease as individuals die / are censored

A total of 21,749 AIDS deaths following ART initiation were reported during 1,783,305 person-years of follow-up time and 6836 deaths (2.5% of patients) occurred in the first six months of ART use. Mean follow-up time was 5.13 years (SD 3.03 years), with 69% of individuals surviving for at least three years following ART initiation.

The mean follow-up time for the 21,124 (7.9%) individuals LTFU was 2.38 years (SD 2.25 years). Of those, 8016 (37.9%) were lost to follow-up within the first year following ART initiation. There were no differences in age, sex or region between those individuals LTFU and those retained (*P* > 0.05 for all); 10.0% of women were LTFU compared with 8.9% of men, the mean age at ART initiation was 38.6 years in those LTFU compared with 37.9 years in those retained and the regional distribution between the two populations was similar.

### Survival analysis

**Fig. 1:**
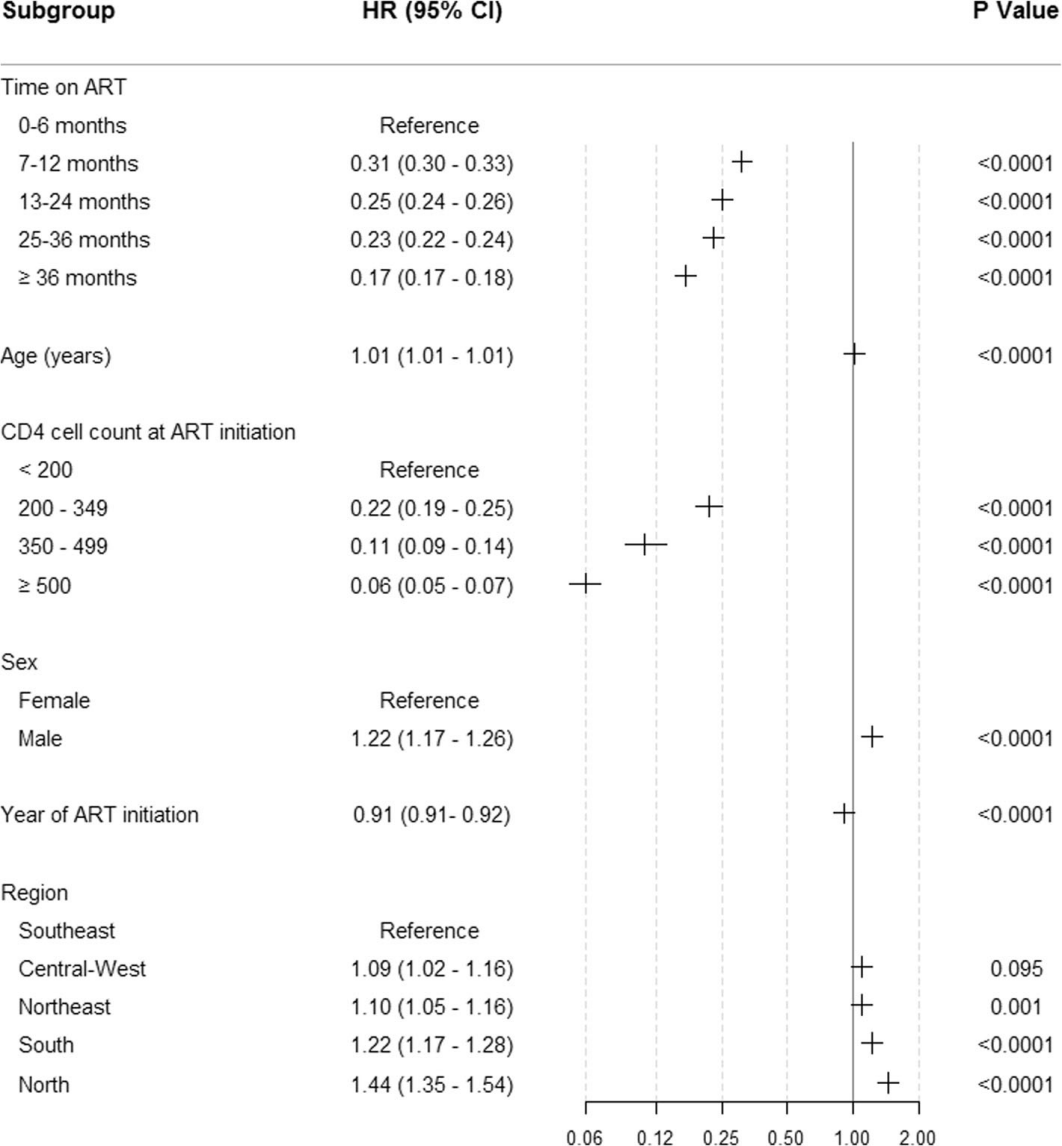
AIDS mortality hazard ratios for baseline characteristics of individuals starting ART. Values presented are the simple main effects with other variables fixed at baseline. X-axis is on the log scale. Data on region are missing for 32 individuals. HR – hazard ratio

All covariates were retained in the final model (age, CD4 cell count, sex, year and region, Additional file 1: Table S1). The risk of dying fell by 69% (HR 0.31, 95% CI 0.30–0.33) after the first six months on ART (Fig. 1). By the end of the first and second years, the hazard ratios fell to 0.25 (0.24–0.26) and 0.23 (0.22–0.24) and after three years on treatment, the hazard ratio was 0.17 (0.17–0.17) relative to the first six months.

Higher CD4 cell counts at ART initiation (CD4 > 500 cells per μL compared with CD4 < 200 cells per μL) reduced the risk of dying by 94% (HR 0.06, 0.05–0.07), and this effect varied by age, sex and region. For example in the Southeast, starting treatment early (CD4 > 500 cells per μL) reduced mortality in the first six months of treatment by 89.1% in men aged 20 years and by 78.5% in men aged 50 compared with starting at CD4 < 200 cells per μL. Similarly for women aged 20 years, mortality rates fell by 90.7 and 81.8% for ages 20 and 50 respectively when treated early (Table 2).

**Table 2:** Annual mortality rates in the first six months of treatment for men and women in the Southeast region starting ART in 2010

CD4 cell count	Age	Male	Female
CD4 ≥ 500	20	0.010 (0.008–0.011)	0.007 (0.006–0.008)
	30	0.013 (0.012–0.015)	0.009 (0.008–0.010)
	40	0.018 (0.017–0.020)	0.013 (0.011–0.014)
	50	0.025 (0.023–0.027)	0.018 (0.016–0.019)
CD4 350–499	20	0.015 (0.013–0.017)	0.012 (0.010–0.013)
	30	0.020 (0.018–0.022)	0.016 (0.014–0.017)
	40	0.026 (0.024–0.028)	0.021 (0.019–0.023)
	50	0.035 (0.032–0.037)	0.027 (0.024–0.030)
CD4 200–349	20	0.026 (0.024–0.028)	0.020 (0.018–0.021)
	30	0.032 (0.030–0.034)	0.024 (0.022–0.026)
	40	0.039 (0.037–0.041)	0.029 (0.028–0.031)
	50	0.048 (0.045–0.050)	0.036 (0.034–0.038)
CD4 < 200	20	0.089 (0.085–0.093)	0.073 (0.069–0.077)
	30	0.097 (0.094–0.101)	0.080 (0.076–0.084)
	40	0.107 (0.103–0.111)	0.088 (0.084–0.091)
	50	0.1117 (0.110–0.122)	0.096 (0.092–0.100)

**Fig. 2:**
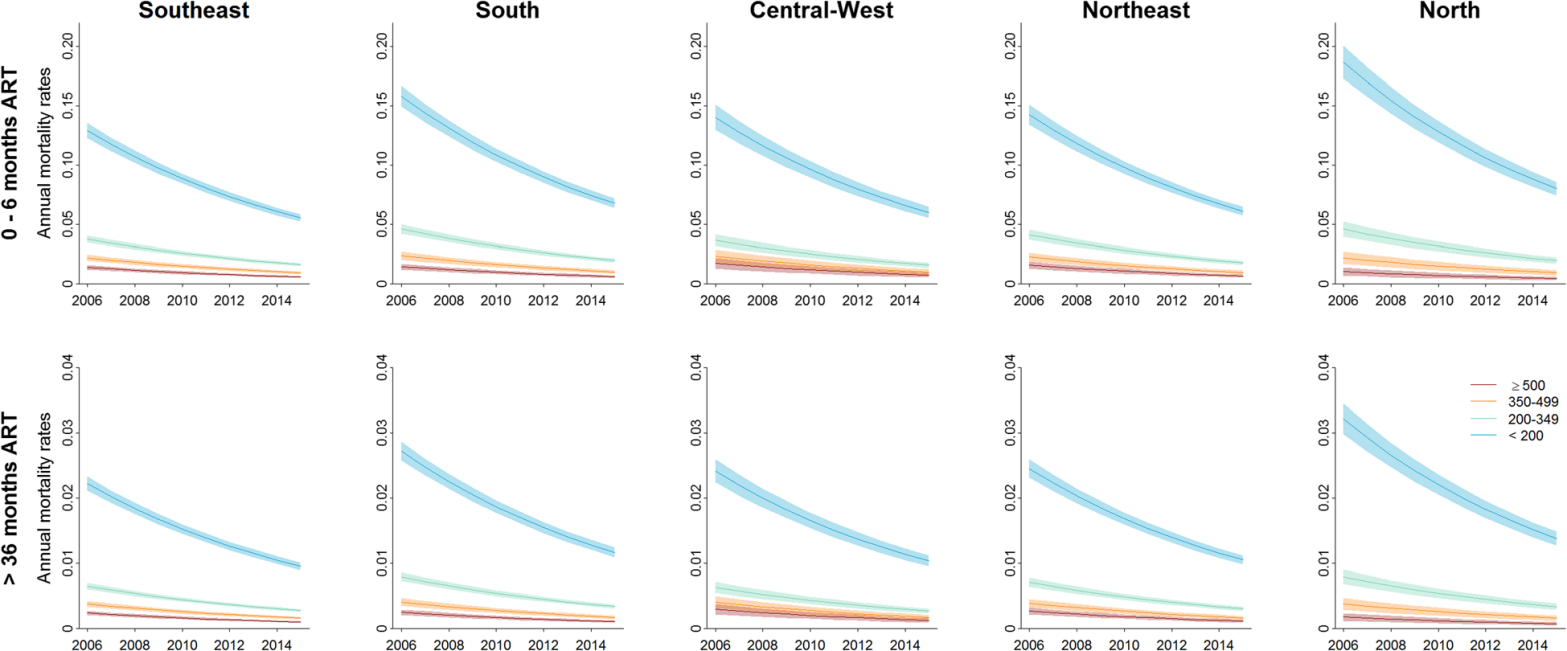
Annual mortality rates over time by CD4 cell count at ART initiation in the first six months of ART (upper panels) and after 36 months on ART (lower panels) for men aged 20. Rates are stratified by the region of residence

Older age, male sex and starting treatment in earlier years were all significantly associated with higher AIDS mortality rates. People living in the North, Northeast and South regions of Brazil had significantly higher mortality rates when starting treatment at CD4 < 200 cells per μL (HR 1.44 [95% CI 1.35, 1.54], 1.10 [1.05–1.16] and 1.22 [1.17–1.28] respectively) than those in the Southeast region (Fig. 2), although this regional effect was diminished when starting ART with higher CD4 cell counts (Additional file 1: Figure S1).

Cross-validation of the model produced a mean accuracy of 0.981 (95% CI 0.980–0.983) indicating high predictive ability. The sensitivity analysis censoring individuals at one year after their last reported observation rather than six months produced similar findings to the main analysis (Additional file 1: Table S2). Broadening the exclusion criteria to include those with CD4 counts within six months prior to ART (rather than three months) added 64,455 individuals to the analysis and again produced similar results (Additional file 1: Table S3). By restricting the analysis to those with over one month of exposure to treatment, the magnitude of the effect of time on treatment decreased (Additional file 1: Table S4).

#### Analysis of risk groups

171,132 individuals (63.5%) had information on risk group (heterosexual transmission, bisexual males, MSM, IDU). Probable transmission due to biological accidents (12 people), haemophilia (82 people) or blood transfusions (124 people) was classified as “other” and represented a very small proportion of cases, mainly occurring in earlier years. The proportion of infections reported via intravenous drug use was low and has been steadily decreasing over time; 1.4% of 36,082 PLHIV starting ART in 2015 were IDUs compared with 6.8% in 2006. The age and sex distribution was similar in those with and without risk group information (median ages 38.4 and 37.9 years respectively, proportion female 0.385 and 0.384). Median CD4 cell counts were slightly higher at ART initiation in people without risk group data (348 cells per μL compared with 328 cells per μL).

**Fig. 3:**
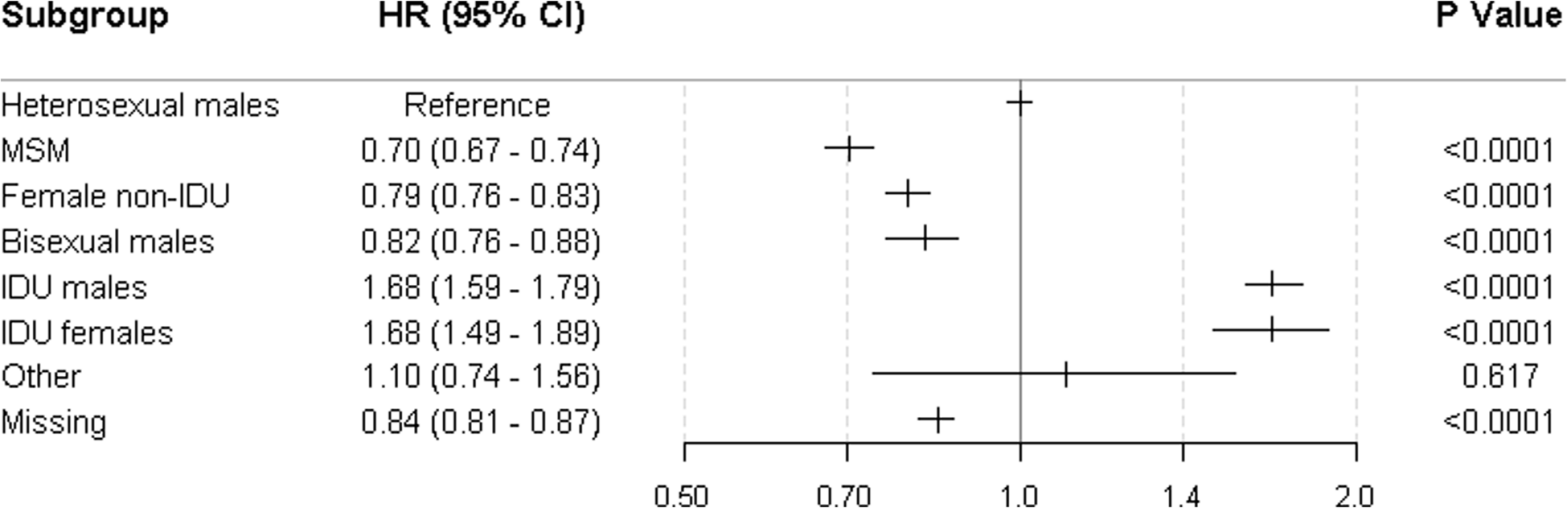
Hazard ratios associated with risk group and adjusted for age, CD4 count at ART initiation, year of initiation, region and time on ART. Interactions between CD4 cell count at ART initiation and age / region were included. X-axis is on the log scale. 98,146 (36%) individuals were missing data on risk. IDU – intravenous drug user; MSM – men who have sex with men

The adjusted hazard ratios showed an increased risk for those reporting intravenous drug use (HR 1.68 [95% CI 1.59–1.79] and 1.68 [1.49–1.89] for males and females) compared with heterosexual males (Fig. 3). Hazard ratios are lower in non-IDU females and MSM with estimates of 0.79 (0.76–0.83) and 0.70 (0.67–0.74) respectively. Individuals missing risk group data had not been registered on the Notifiable Diseases Database and as expected, have a lower hazard of death (0.84 [0.81–0.87]). No differences were detected between heterosexual males and the risk group “others”. The effects of all other predictor variables remained similar to the main model (Additional file 1: Table S5).

## Discussion

Early ART initiation (amongst patients with CD4 cell counts ≥500 cells per μL) was associated with a 94% reduction in the risk of AIDS death during the first six months compared with starting at < 200 cells per μL. Older age or a history of intravenous drug use resulted in higher mortality rates consistent with earlier studies. [1, 12, 15, 27]

The poorer survival in the North may reflect differences in access to healthcare and is concerning as coinciding increases in incidence rates have also been reported in this region. [13, 14] Many of the larger, specialised services are in the Southeast, which has historically borne the greatest burden of the HIV epidemic. [28] In the large northern states, fewer clinics offer specialised care and patients may be at greater risk of non-adherence.

The relative effects of early treatment on survival were consistent after adjustments for other risk factors for mortality but the size of the effect varies by age, sex and region. The survival benefit from starting treatment early is greatest in women, younger ages and in the North region. Increasingly smaller gains are seen as age at ART initiation increases, with relative reductions in mortality rates of 78.4 and 81.8% in men and women aged 50 years starting treatment early (CD4 cell counts at ART initiation ≥500 versus < 200 cells per μL) compared with 89.1 and 90.7% in 20 year olds. When excluding individuals with less than one month of exposure to ART, there was a smaller impact of time on treatment relative to the first 1–6 months. This is likely due to the high mortality observed in this initial period, before viral loads are reduced by treatment.

Vital status is reported from the Mortality Information System database which has an estimated coverage of over 95% in 2000–2010 although misclassification of AIDS deaths may result in an underestimation of mortality rates. [29] Regional variations in misclassification of deaths also likely exist but to our knowledge, have not been well studied. [30] The majority of those lost to follow up are in recent years (68% of individuals LTFU are from 2012 onwards, see Supplementary Fig. 2). Patients classified as LTFU may still have been accessing care in the private sector despite failing to adhere to ART, which would not be reported in the Ministry of Health’s surveillance databases. Given the low rates of LTFU here (7.9%) coupled with the near completeness of linked vital registration data, we did not apply any additional correction factors to compensate for possible under-reporting of deaths.

We ignored co-morbidities and changes or interruptions in treatment, opting instead to provide generalised estimates which broadly take adherence patterns into account. Coverage of the ART dispensation system has been variable throughout the country and over time, but has remained consistently high (> 90%) between 2006 and 2015. [31] This is unlikely to significantly impact our findings here but may lead to under-estimations of the amount of time on ART for some people and bias our estimates of treatment impact upwards. The majority of those who initiated ART but were excluded from this analysis were missing CD4 cell count measurements. These individuals may have had poorer engagement with the health care system and may subsequently experience a worse prognosis.

Data on risk group are self-reported via the case-reporting surveillance system which records predominantly AIDS cases and may be misreported due to perceived stigma. The lower mortality rates in those missing information on risk group may be due to the absence of clinical symptoms which prompt individuals to seek healthcare and consequently be reported via this route. Additionally, case-reporting is lower in private healthcare facilities. The lower mortality rates amongst those without case-reports may reflect the fact that a greater proportion of them were being followed by private services. However, we expect the relative findings of highest survival in females and poorest survival in IDUs to remain applicable.

A key strength of this study is the use of real program data from three independent surveillance networks linked with vital registration. As these results do not rely on cohorts of individuals selected for inclusion or on data obtained from single sites, they will be broadly representative of the general population of PLHIV on ART in Brazil. We do not consider viral load here which could also be used as a predictor of mortality. An interesting next step could be the inclusion of both CD4 cell counts and viral load using Principal Component Analysis. [32]

The availability of early treatment in Brazil has led to increased linkage, higher median CD4 counts at ART initiation and 88% viral suppression in those treated [10]. Together with earlier studies demonstrating a significant reduction in mortality [8, 33, 34], lower incidence of AIDS-related and non-AIDS-related events [35] and a reduced risk of sexual transmission with early ART use [19, 36], these results provide further evidence supporting the implementation of early and lifelong ART in line with Brazilian and UNAIDS guidelines. [37] There is a need to evaluate the incidence of adverse effects resulting from the increased longer-term exposure to ART and the impact of early treatment initiation on long-term adherence, especially among high-risk populations.

Despite huge progress in early diagnosis and linkage to care, many vulnerable people at high risk are still not benefitting from early treatment and some of those, such as IDUs, experience disproportionately high mortality rates. In addition, 47.2% of those who died of AIDS-related causes between 2009 and 2013 had never accessed ART. [38] Brazil’s goal to end the AIDS epidemic by 2030 requires focussing prevention and treatment in these high-risk groups.

## Conclusions

This study highlighted the disparities in mortality rates experienced by many PLHIV in Brazil due to age, sex, risk behaviour and geographical location. We showed the increased risk of mortality in males compared with females, in older age groups, those starting treatment at low CD4 counts and those living in regions outside the South-East or Central-West of Brazil. Starting treatment early (CD4 cell counts ≥500 cells per μL) significantly reduced the risk of AIDS death in the first six months of treatment and this survival benefit is greatest in women, younger age groups and people living in the North region. This study further emphasizes the benefits of early and lifelong treatment in PLHIV, a policy which has been implemented in Brazil since 2014, but many vulnerable people are still failing to access treatment early and experiencing unacceptably high AIDS mortality rates.

## Additional file

Additional file 1: Additional tables and figures to support the main article. (DOCX 2763 kb)

## Abbreviations

ART: antiretroviral therapy
DIAHV: Department of STI, HIV/AIDS and Viral Hepatitis
HR: hazard ratio
IDU: intravenous drug user
IQR: inter-quartile range
LTFU: lost to follow-up
MSM: men who have sex with men
PLHIV: people living with HIV
