# Supplementary material for: Determinants of survival of people living with HIV/AIDS on antiretroviral therapy in Brazil 2006–2015
Source: BMC Infect Dis. 2019 Feb 28;19:206. doi: 10.1186/s12879-019-3844-3 (PMC6396460; doi:10.1186/s12879-019-3844-3)
Supplement: Supplementary file 1 — Additional tables and figures to support the main article. (DOCX 2763 kb) [file 12879_2019_3844_MOESM1_ESM.docx]

**SUPPLEMENTARY INFORMATION**

Table S1. Complete output from the piecewise exponential regression analysis presented in the main text including interactions.

| Variable | Estimate | Standard Error | P value |
| --- | --- | --- | --- |
| (Intercept) | 186.000 | 5.655 | <0.0001 |
|  |  |  |  |
| Time on ART |  |  |  |
| 0 – 6 months | Reference |  |  |
| 7 –12 months | -1.156 | 0.025 | <0.0001 |
| 13 –24 months | -1.385 | 0.022 | <0.0001 |
| 25 – 36 months | -1.469 | 0.023 | <0.0001 |
| > 36 months | -1.760 | 0.018 | <0.0001 |
|  |  |  |  |
|  |  |  |  |
| Age (years) | 0.009 | 0.001 | <0.0001 |
|  |  |  |  |
| CD4 cell count at ART initiation |  |  |  |
| CD4 < 200 | Reference |  |  |
| CD4 200 – 349 | -1.530 | 0.072 | <0.0001 |
| CD4 350 – 499 | -2.175 | 0.104 | <0.0001 |
| CD4 ≥ 500 | -2.832 | 0.117 | <0.0001 |
|  |  |  |  |
| Sex |  |  |  |
| Female | Reference |  |  |
| Male | 0.197 | 0.018 | <0.0001 |
|  |  |  |  |
| Year of ART initiation | -0.094 | 0.003 | <0.0001 |
|  |  |  |  |
| Region |  |  |  |
| Southeast | Reference |  |  |
| Central-West | 0.082 | 0.034 | 0.015 |
| North | 0.367 | 0.033 | <0.0001 |
| Northeast | 0.098 | 0.025 | <0.0001 |
| South | 0.202 | 0.021 | <0.0001 |
|  |  |  |  |
| Interactions |  |  |  |
|  |  |  |  |
| CD4 200 – 349 x age | 0.011 | 0.002 | <0.0001 |
| CD4 350 – 499 x age | 0.018 | 0.002 | <0.0001 |
| CD4 ≥ 500 x age | 0.023 | 0.002 | <0.0001 |
|  |  |  |  |
| CD4 200 – 349 x sex (male) | 0.089 | 0.036 | 0.013 |
| CD4 350 – 499 x sex (male) | 0.050 | 0.053 | 0.344 |
| CD4 ≥ 500 x sex (male) | 0.165 | 0.059 | 0.005 |
|  |  |  |  |
| CD4 200 – 349 x region (Central-West) | -0.112 | 0.073 | 0.128 |
| CD4 350 – 499 x region (Central-West) | -0.020 | 0.114 | 0.860 |
| CD4 ≥ 500 x region (Central-West) | 0.130 | 0.132 | 0.323 |
|  |  |  |  |
| CD4 200 – 349 x region (North) | -0.174 | 0.074 | 0.018 |
| CD4 350 – 499 x region (North) | -0.366 | 0.120 | 0.0024 |
| CD4 ≥ 500 x region (North) | -0.660 | 0.163 | <0.0001 |
|  |  |  |  |
| CD4 200 – 349 x region (Northeast) | -0.010 | 0.052 | 0.855 |
| CD4 350 – 499 x region (Northeast) | -0.069 | 0.079 | 0.380 |
| CD4 ≥ 500 x region (Northeast) | 0.019 | 0.087 | 0.832 |
|  |  |  |  |
| CD4 200 – 349 x region (South) | -0.003 | 0.041 | 0.943 |
| CD4 350 – 499 x region (South) | -0.127 | 0.064 | 0.047 |
| CD4 ≥ 500 x region (South) | -0.161 | 0.076 | 0.035 |

Abbreviations: ART antiretroviral therapy.


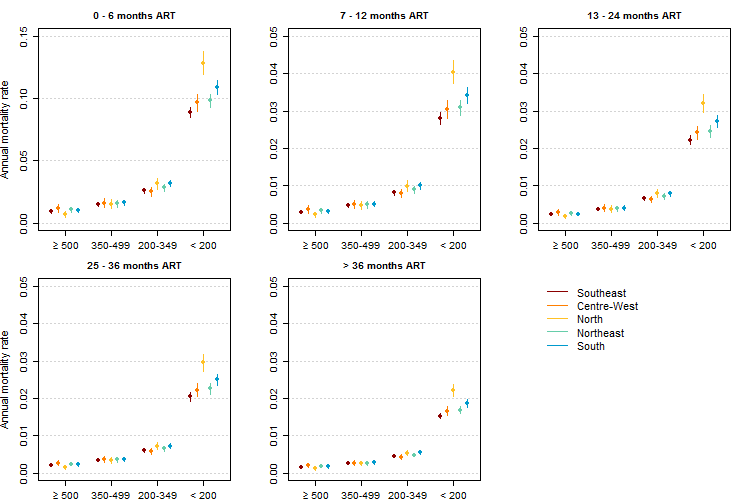


Supplementary Figure 1. Annual mortality rates by time on treatment, CD4 cell count at ART initiation and region for men aged 20 starting treatment in 2010. Abbreviations: ART antiretroviral therapy.

Supplementary Table S2. Sensitivity analysis – abridged results showing the main effects from the piecewise exponential model with individuals censored one year after their last observations.

| Variable | Estimate | Std Error | P value |
| --- | --- | --- | --- |
| (Intercept) | 213.200 | 5.570 | <0.0001 |
|  |  |  |  |
| Time on ART |  |  |  |
| 0 – 6 months | Reference |  |  |
| 7 –12 months | -1.175 | 0.025 | <0.0001 |
| 13 –24 months | -1.411 | 0.022 | <0.0001 |
| 25 – 36 months | -1.523 | 0.023 | <0.0001 |
| > 36 months | -1.897 | 0.017 | <0.0001 |
|  |  |  |  |
|  |  |  |  |
| Age (years) | 0.009 | 0.001 | <0.0001 |
|  |  |  |  |
| CD4 cell count at ART initiation |  |  |  |
| CD4 < 200 | Reference |  |  |
| CD4 200 – 349 | -1.529 | 0.072 | <0.0001 |
| CD4 350 – 499 | -2.181 | 0.104 | <0.0001 |
| CD4 ≥ 500 | -2.844 | 0.117 | <0.0001 |
|  |  |  |  |
| Sex |  |  |  |
| Female | Reference |  |  |
| Male | 0.196 | 0.018 | <0.0001 |
|  |  |  |  |
| Year of ART initiation | -0.108 | 0.003 | <0.0001 |
|  |  |  |  |
| Region |  |  |  |
| Southeast | Reference |  |  |
| Central-West | 0.083 | 0.034 | 0.015 |
| North | 0.369 | 0.033 | <0.0001 |
| Northeast | 0.100 | 0.025 | <0.0001 |
| South | 0.202 | 0.021 | <0.0001 |

Abbreviations: ART antiretroviral therapy.

Supplementary Table S3. Sensitivity analysis – abridged results showing the main effects from the piecewise exponential model including those with CD4 cell counts up to six months prior to ART initiation (an additional 64,548 individuals, total 333,624).

| Variable | Estimate | Std Error | P value |
| --- | --- | --- | --- |
| (Intercept) | 186.300 | 5.114 | <0.0001 |
|  |  |  |  |
| Time on ART |  |  |  |
| 0 – 6 months | Reference |  |  |
| 7 –12 months | -1.051 | 0.023 | <0.0001 |
| 13 –24 months | -1.250 | 0.020 | <0.0001 |
| 25 – 36 months | -1.316 | 0.021 | <0.0001 |
| > 36 months | -1.607 | 0.016 | <0.0001 |
|  |  |  |  |
|  |  |  |  |
| Age (years) | 0.008 | 0.001 | <0.0001 |
|  |  |  |  |
| CD4 cell count at ART initiation |  |  |  |
| CD4 < 200 | Reference |  |  |
| CD4 200 – 349 | -1.472 | 0.063 | <0.0001 |
| CD4 350 – 499 | -2.129 | 0.089 | <0.0001 |
| CD4 ≥ 500 | -2.862 | 0.097 | <0.0001 |
|  |  |  |  |
| Sex |  |  |  |
| Female | Reference |  |  |
| Male | 0.175 | 0.017 | <0.0001 |
|  |  |  |  |
| Year of ART initiation | -0.094 | 0.003 | <0.0001 |
|  |  |  |  |
| Region |  |  |  |
| Southeast | Reference |  |  |
| Central-West | 0.044 | 0.032 | 0.164 |
| North | 0.342 | 0.031 | <0.0001 |
| Northeast | 0.082 | 0.023 | 0.0003 |
| South | 0.166 | 0.020 | <0.0001 |

Abbreviations: ART antiretroviral therapy.

Supplementary Table S4. Sensitivity analysis – abridged results showing the main effects from the piecewise exponential model excluding those who contributed less than one month of person-time (2714 individuals excluded, total 266,414).

| Variable | Estimate | Std Error | P value |
| --- | --- | --- | --- |
| (Intercept) | 231.800 | 6.215 | <0.0001 |
|  |  |  |  |
| Time on ART |  |  |  |
| 1 – 6 months | Reference |  |  |
| 7 –12 months | -0.653 | 0.027 | <0.0001 |
| 13 –24 months | -0.885 | 0.024 | <0.0001 |
| 25 – 36 months | -0.977 | 0.025 | <0.0001 |
| > 36 months | -1.301 | 0.020 | <0.0001 |
|  |  |  |  |
|  |  |  |  |
| Age (years) | 0.007 | 0.001 | <0.0001 |
|  |  |  |  |
| CD4 cell count at ART initiation |  |  |  |
| CD4 < 200 | Reference |  |  |
| CD4 200 – 349 | -1.532 | 0.075 | <0.0001 |
| CD4 350 – 499 | -2.120 | 0.108 | <0.0001 |
| CD4 ≥ 500 | -2.812 | 0.121 | <0.0001 |
|  |  |  |  |
| Sex |  |  |  |
| Female | Reference |  |  |
| Male | 0.165 | 0.020 | <0.0001 |
|  |  |  |  |
| Year of ART initiation | -0.117 | 0.003 | <0.0001 |
|  |  |  |  |
| Region |  |  |  |
| Southeast | Reference |  |  |
| Central-West | 0.035 | 0.037 | 0.353 |
| North | 0.339 | 0.037 | <0.0001 |
| Northeast | 0.060 | 0.027 | 0.028 |
| South | 0.162 | 0.023 | <0.0001 |

Abbreviations: ART antiretroviral therapy.


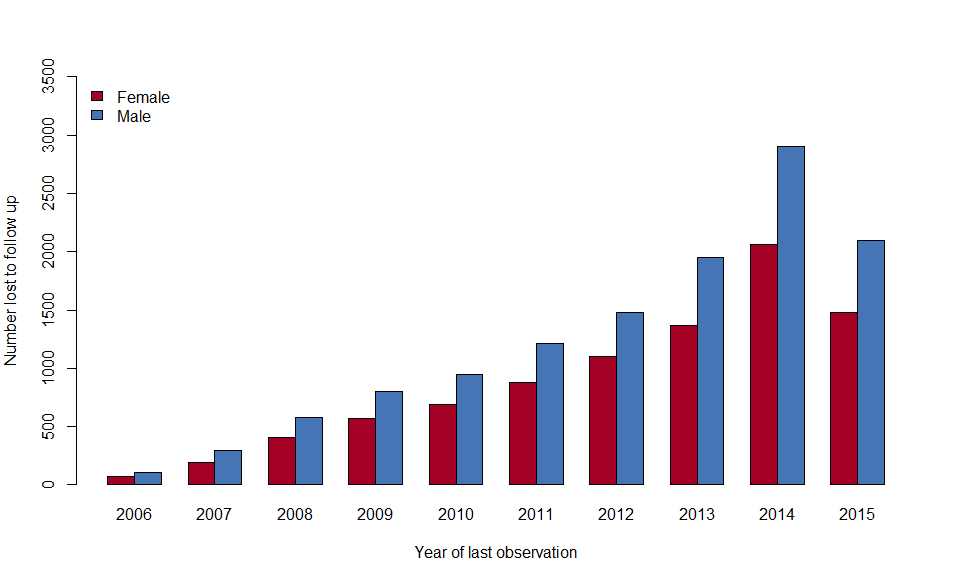


Supplementary Figure 2. Number of individuals lost to follow up according to the year of their last recorded observation, stratified by sex. Numbers lost to follow-up: 8,784 females (8.5% of total 103,967) and 12,340 males (7.4% of total 166,214).

Supplementary Table S5. Complete output from the piecewise exponential regression analysis including risk groups presented in the main text.

| Variable | Estimate | Standard Error | P value |
| --- | --- | --- | --- |
| (Intercept) | 169.100 | 5.703 | <0.0001 |
|  |  |  |  |
| Risk group |  |  |  |
| Heterosexual males | Reference |  |  |
| MSM | -0.350 | 0.027 | <0.0001 |
| Female non-IDU | -0.230 | 0.020 | <0.0001 |
| Bisexual males | -0.197 | 0.036 | <0.0001 |
| IDU males | 0.521 | 0.030 | <0.0001 |
| IDU females | 0.519 | 0.060 | <0.0001 |
| Other | 0.095 | 0.190 | 0.617 |
| Missing | -0.170 | 0.018 | <0.0001 |
|  |  |  |  |
| Time on ART |  |  |  |
| 0 – 6 months | Reference |  |  |
| 7 –12 months | -1.155 | 0.025 | <0.0001 |
| 13 –24 months | -1.384 | 0.022 | <0.0001 |
| 25 – 36 months | -1.466 | 0.023 | <0.0001 |
| > 36 months | -1.754 | 0.018 | <0.0001 |
|  |  |  |  |
|  |  |  |  |
| Age (years) | 0.009 | 0.001 | <0.0001 |
|  |  |  |  |
| CD4 cell count at ART initiation |  |  |  |
| CD4 < 200 | Reference |  |  |
| CD4 200 – 349 | -1.460 | 0.068 | <0.0001 |
| CD4 350 – 499 | -2.142 | 0.099 | <0.0001 |
| CD4 ≥ 500 | -2.770 | 0.113 | <0.0001 |
|  |  |  |  |
| Year of ART initiation | -0.085 | 0.003 | <0.0001 |
|  |  |  |  |
| Region |  |  |  |
| Southeast | Reference |  |  |
| Central-West | 0.092 | 0.034 | 0.007 |
| North | 0.375 | 0.033 | <0.0001 |
| Northeast | 0.125 | 0.025 | <0.0001 |
| South | 0.161 | 0.021 | <0.0001 |
|  |  |  |  |
| Interactions |  |  |  |
|  |  |  |  |
| CD4 200 – 349 x age | 0.011 | 0.002 | <0.0001 |
| CD4 350 – 499 x age | 0.018 | 0.002 | <0.0001 |
| CD4 ≥ 500 x age | 0.024 | 0.002 | <0.0001 |
|  |  |  |  |
| CD4 200 – 349 x region (Central-West) | -0.116 | 0.073 | 0.114 |
| CD4 350 – 499 x region (Central-West) | -0.028 | 0.114 | 0.808 |
| CD4 ≥ 500 x region (Central-West) | 0.136 | 0.132 | 0.301 |
|  |  |  |  |
| CD4 200 – 349 x region (North) | -0.184 | 0.074 | 0.013 |
| CD4 350 – 499 x region (North) | -0.368 | 0.120 | 0.002 |
| CD4 ≥ 500 x region (North) | -0.646 | 0.163 | <0.0001 |
|  |  |  |  |
| CD4 200 – 349 x region (Northeast) | -0.020 | 0.052 | 0.700 |
| CD4 350 – 499 x region (Northeast) | -0.079 | 0.079 | 0.317 |
| CD4 ≥ 500 x region (Northeast) | 0.019 | 0.087 | 0.827 |
|  |  |  |  |
| CD4 200 – 349 x region (South) | -0.012 | 0.040 | 0.774 |
| CD4 350 – 499 x region (South) | -0.128 | 0.064 | 0.044 |
| CD4 ≥ 500 x region (South) | -0.160 | 0.076 | 0.035 |

Abbreviations: ART antiretroviral therapy, MSM men who have sex with men, IDU intravenous drug users.
